# Assessment of high sensitivity C-reactive protein and coronary plaque characteristics by computed tomography in patients with and without diabetes mellitus

**DOI:** 10.1186/s12872-020-01704-w

**Published:** 2020-10-07

**Authors:** Hai-Ting Zhou, De-Li Zhao, Guo-Kun Wang, Tian-Zuo Wang, Hong-Wei Liang, Jin-Ling Zhang

**Affiliations:** Department of CT, The Second Affiliated Hospital of Harbin Medical University, Harbin, 150086 China

**Keywords:** Diabetes, CT, X-ray, Coronary Angiography

## Abstract

**Background:**

To evaluate the coronary plaque characteristics of coronary arteries using computed tomography angiography (CTA) in order to assess the risk of coronary artery disease and the relevance of high sensitivity C reactive protein (hs-CRP) in patients with Diabetes Mellitus (DM).

**Methods:**

The clinical data of 400 DM patients and 400 non-DM patients from January 2017 to December 2019 were collected, including the results of coronaryCTA. The plasma hs-CRP level of the two groups were divided into three groups: CRP ≤ 1, 1 < CRP ≤ 2, CRP > 2. The correlation of the degree of stenosis, the number of plaques, the nature of plaques and hs-CR*P* value between the two groups was evaluated.

**Results:**

Compared with non-DM patients, the incidence of coronary artery plaques and lumen stenosis in DM patients was more higher than that in non-DM patients. DM patients were more likely to have more diseased vessels, especially diffuse vascular disease (12.00% vs 1.75%; *P* < 0.001). Subjects with high hs-CRP levels were more likely to have any plaque compared with individuals showing normal hs-CRP levels (*p*<0.01). There was no statistical significance in non calcified plaque with high level of hs-CRP, but the occurrence of plaque types in DM group was statistically significant compared with other hs-CRP levels in non DM group. Subjects with high hs-CRP were observed to be at increased risk for the presence of calcified plaque and severe narrowing in the unadjusted values.

**Conclusions:**

Coronary CTA combined with hs-CRP can accurately detect the characteristics of coronary artery stenosis and plaque in DM patients, which has an important clinical value in the risk assessment of coronary heart disease in DM patients.

## Background

Coronary artery disease (CAD) is a commonly observed heart complication in patients with diabetes (DM). CAD is frequently developed when DM progresses [1, 2]. Furthermore, CAD is detected in as many as 50% of patients with DM. As a leading cause of death in patients with DM, combination of CAD and DM is also one of the major risk factors for acute coronary incidents [3, 4]. Multi slice computed tomography (MSCT) is currently used as an effective and non-invasive method for diagnosing coronary artery disease [5].

Inflammation is a fundamental component of atherosclerosis [6]. hs-CRP can promote inflammation and atherosclerosis. hs-CRP is a non-specific inflammatory marker. The higher the level of hs-CRP, the greater the risk of acute myocardial infarction (AMI). However, studies on CAD population have found that hs-CRP level is positively correlated with the severity of coronary artery disease [6, 7]. Moreover, elevated hs-CRP and coronary artery specific plaque subtypes were associated with poor prognosis [8]. Since hs-CRP has been shown to play a major role in the development of atherosclerosis in DM patients, Understanding how CRP and vulnerable plaques are related, and using imaging techniques to assess this relationship may enable the early identification of vulnerable patients. This study used CTA to examine DM patients and investigate the association between hs-CRP, coronary plaque characteristics, and stenosis of the vascular lumen.

## Methods

### Study population

From January 2017 and December 2019, a total of 800 consecutive asymptomatic patients from endocrinology and cardiology department who had undergone coronary CTA were retrospectively enrolled into DM group (*n* = 400) and non-DM group (*n* = 400). They were 411 men and 389 women of 56.7 ± 12.1 years old, with body mass index (BMI) of 22.27 ± 2.41 kg/m^2^. The average age of onset was 8.4 ± 6.7 years. The clinical data of patients included age, sex, BMI, heart rate, hypertension, smoking history and family history of coronary disease. Laboratory results included triglyceride, total cholesterol, low-density lipoprotein cholesterol, high-density lipoprotein-cholesterol and hs-CRP. Serum hs-CRP levels were measured before coronary CTA. The diagnostic criteria for DM were based on WHO guidelines in 1999, that is diabetic symptoms plus: a random intravenous plasma glucose concentration ≥ 11.1 mmol/l, or fasting blood glucose concentration ≥ 7.0 mmol/l (whole blood ≥6.1 mmol/l), or two-hour blood glucose concentration ≥ 11.1 mmol/l and 75 g anhydrous glucose after 2 hours of oral glucose tolerance test (OGTT). The study was approved by the Institutional Review Board of the hospital, and informed consent was obtained from each patient.

### CT protocols and scanning method

Coronary CTA examination was performed using a 256-slice CT (Revolution CT; GE Healthcare, Milwaukee, WI, USA). The scan protocols were as follows: detector width was160 mm; gantry rotation time was 0.28 s; time resolution was 29 ms; tube voltage was 100 kVp, the tube current is set according to the machine’s automatic recommendation. The contrast material (Ultravist Solution 350 mg I/mL; Bayer Healthcare, Berlin, Germany) was intravenously injected through an antecubital vein using a 20-gauge needle connected to a power injector (SCT-211; Medrad Inc., Indianola, PA, USA). A total amount of 60–80 ml of contrast material was injected at 4.5–5.0 ml/s followed by 30 ml of saline chaser. Retrospective electrocardiographic gating was used to eliminate cardiac motion artefacts. Data acquisition was completed within 0.7 s.

### Biochemical indicators

A total of 6 ml of blood sample was collected from each patient in the early morning of the second day (prior to food intake), following admission to the hospital. Plasma was isolated within 1 hour after blood collection and plasma hs-CRP content was measured by absorption (OD value) readout using spectro photometry (Bio-RAD model 608, USA). All the biochemical indexes were finished by the hospital inspection center.

### Evaluation criteria for coronary artery stenosis and plaques characteristics

**Fig. 1:**
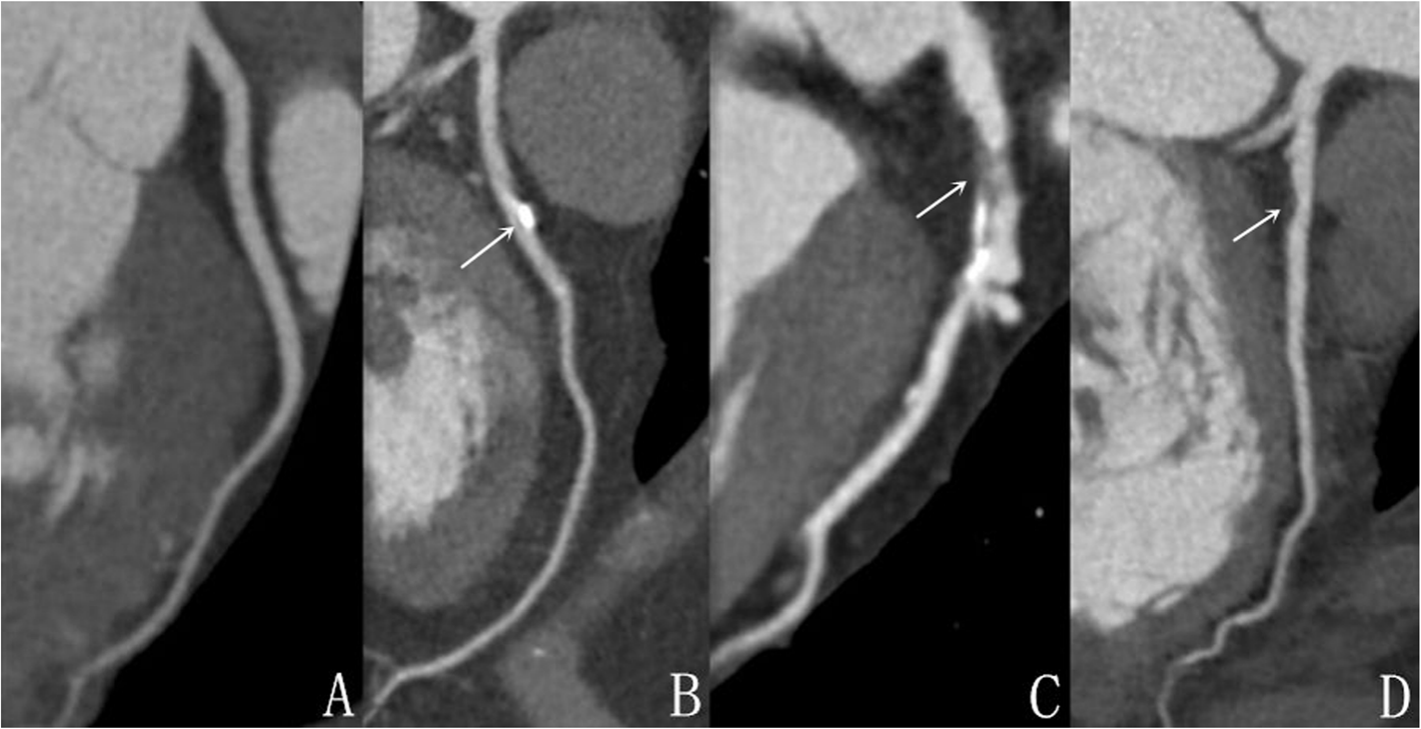
Different types of coronary artery plaque. **a** is normal coronary artery, **b** is calcified plaque, **c** is mixed plaque and **d** is non-calcified plaque. As shown by the arrow in the figure

**Fig. 2:**
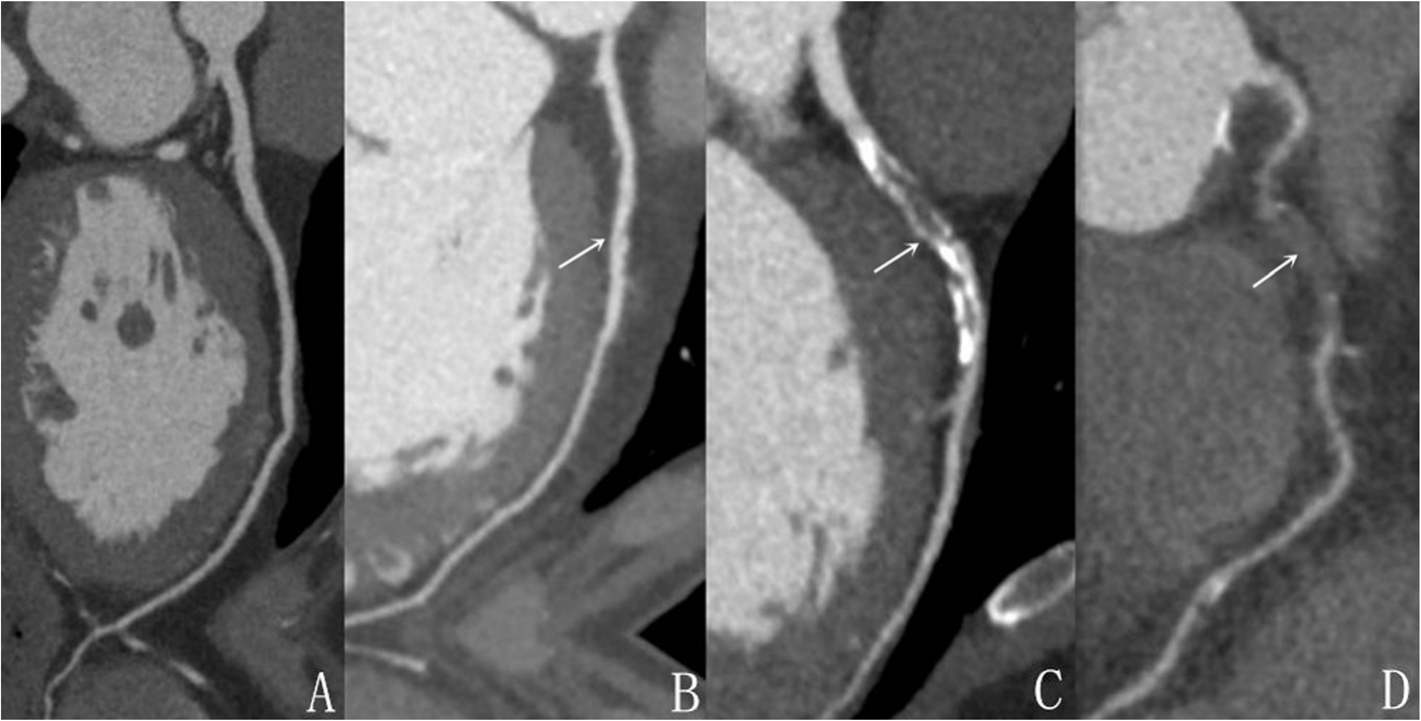
Different degrees of coronary stenosis. **a** is normal coronary artery, **b** is mild stenosis, **c** is moderate stenosis, **d** is severe stenosis or occlusion. As shown by the arrow in the figure

Two radiologists, with 3–5 years of radiology experience, were recruited to evaluate the images in a double-blinded manner. When the two radiologists expressed differing opinions, they analyzed the images together and re-evaluated their initial assessment until an agreement was reached. The assessment of coronary artery stenosis is based on curved planar reformation (CPR) reconstruction images. The number of affected coronaries is divided into single, double, triple, and diffuse lesions. The judgment of coronary artery stenosis is based on the visual diameter assessment method commonly used internationally: The degree of vascular stenosis (Target reference vessel diameter (RVD)–minimal luminal diameter) / RVD × 100%. The degree of coronary narrowness is categorized into four levels: 1) mild, narrow vascular lumen (< 50%), 2) moderate, narrow vascular lumen (50–70%), 3) severe, narrow vascular lumen (> 75%), and 4) completely closed, narrow vascular lumen (100%). The classification of coronary plaques was based upon the Schroeder standard in CT value [9]: Non-calcified plaques: Negative 42–47 HU; mixed plaques 61–112 HU; calcified plaques 126–736 HU. The lowest CT value was selected from randomly chosen four or more sites in the area of interest (> 1.0 cm^2^) in the axis or CPR image (Figs. 1 and 2).

### Statistical analysis

All statistical analysis was conducted using SAS 9.3 software. The quantitative data followed the normal distribution by the mean and the standard deviation, but it deviated from normal distribution when the median and the upper and lower quartiles were used. For counting purposes, frequency and percentage values were measured and a Chi-squared test was used to compare between groups, while Fisher method was applied when criteria for Chi-squared test were not met. For leveling purposes, frequency and percentage values were measured and Wilcoxon rank-sum test was used to compare groups. When *p* < 0.05, a statistically significant difference was inferre. Kappa test was used to assess the consistency between the two radiologists’ readings.

## Results

### Baseline characteristics

Compared with subjects non-DM, no significant differences in age, BMI, mean heart ratein and the levels of total cholesterol, triglyceride, high-density lipoprotein cholesterol, and low-density lipoproteincholesterol were obtained. In medication use, compared with non-DM, oral hypoglecemic agents and aspirin had significant statistical significance, while beta-blocker, ACE inhibitor or ARB and calcium channel blockers had no significant difference. The serum level of hs-CRP in DM group was significantly higher than that in non DM group [1.785 (0.88–4.71) mg/L vs. 1.08 (0.53–2.7) mg/L, *p* < 0.05]. Table 1 summarizes the general characteristics of the study population.

**Table 1:** Basic characteristics of all the patients

Characteristic	All patients (*n* = 800)	DM2 patients (*n* = 400)	Non-DM patients (*n* = 400)
Age (years)	56.7 ± 12.1	55.4 ± 13.5	56.3 ± 10.8
Male (%)	411 (51.37)	216 (54.00)	195 (48.75)
BMI (kg/m2)	22.27 ± 2.41	22.43 ± 2.16	21.85 ± 2.32
Mean heart rate	62.7 ± 11.4	64.3 ± 12.5	61.8 ± 13.1
Hypertension (%)	510 (63.75)	297 (74.25)	213 (53.25)
Current smoking (%)	343 (42.88)	145 (36.25)	198 (49.50)
Family history of coronary disease (%)	484 (60.50)	263 (65.75)	221 (55.25)
CIMT (mm)	0.83 ± 0.22	0.96 ± 0.31	0.78 ± 0.15
TC (mmol/L)	4.77 ± 1.14	4.70 ± 1.12	4.85 ± 1.13
TG (mmol/L)	2.15 ± 1.78	2.41 ± 2.03	1.88 ± 1.45
HDL-C (mmol/L)	1.27 ± 0.31	1.22 ± 0.30	1.33 ± 0.31
LDL-C (mmol/L)	2.73 ± 0.88	2.65 ± 0.84	2.82 ± 0.90
hs-CRP (mmol/L)	1.45 (0.65–3.405)	1.785 (0.88–4.71)	1.08 (0.53–2.7)
Medication use			
Oral hypoglecemic agents(%)	421 (52.63)	268 (67.00)	153 (38.25)
Aspirin(%)	324 (40.50)	209 (52.25)	115 (28.75)
Beta-blocker (%)	139 (17.38)	72 (18.00)	67 (16.75)
ACE inhibitor or ARB(%)	157 (19.63)	83 (20.75)	74 (18.50)
Calcium channel blockers(%)	143 (17.88)	74 (18.50)	69 (17.25)

Data are mean ± SD or n (%)

*BMI* body-mass index, *CIMT* carotid intima-media thickness, *TC* total Cholesterol, *TG* triglyceride, *HDL-C* high-density lipoprotein cholesterol, *LDL-C* low-density lipoprotein cholesterol, *ACE* angiotensin converting enzyme inhibitors, *ARB* angiotensin receptor blockers

### CTA data analysis between DM and non-DM patients

CTA imaging of all patients was successfully completed and satisfactory consistency was achieved from two radiologists (Kappa = 0.87). More calcified plaques, mixed Plaque and less non calcified plaques were detected in DM compared with non-DM patients, respectively (67.25% vs. 52.00%, *P* < 0.0001; 59.25% vs. 39.00%, *P* < 0.0001; 79.25% vs. 68.75%, *P* = 0.0001). More moderate narrowing, severe narrowing and less mild narrowing were detected in DM compared with non-DM patients, respectively (37.00% vs. 20.50%, *P* < 0.0001; 22.75% vs. 8.75%, *P* < 0.0001; 95.75% vs. 96.25%, *P* = 0.7182). Patients with DM are prone to develop multiple vascular lesions. In this study, more diseased vessels were found in patients with DM compared with non-DM, whether single vessel, double vessel, three vessel and diffuse distribution, especially diffuse vessel disease was more commonly detected in DM patients than non-DM patients (12.00% vs 1.75%; *p* < 0.001). The plaque burden, stenosis data and the number of vessels are shown in Tables 2 and 3.

**Table 2:** Comparison of the plaque burden of coronary artery plaque and the degree of coronary artery stenosis between DM and non-DM patients

	DM	Non-DM	χ2 Value	*P* Value
Plaque burden				
Calcified plaque	269 (67.25%)	208 (52.00%)	19.321	< 0.0001
Mixed plaque	237 (59.25%)	156 (39.00%)	32.815	< 0.0001
Non-calcified plaque	317 (79.25%)	275 (68.75%)	11.4605	0.0007
Grading of stenosis				
Mild stenosis	383 (95.75%)	385 (96.25%)	0.1302	0.7182
Moderate stenosis	148 (37.00%)	82 (20.50%)	26.5812	< 0.0001
Severe or occlusion	91 (22.75%)	35 (8.75%)	29.5417	< 0.0001

**Table 3:** Comparison of the number of vessels with coronary plaques between DM and non-DM patients

	DM	Non-DM
SIngle vessels	109 (27.25%)	181 (45.25%)
Double vessels	107 (26.75%)	124 (31.00%)
Triple vessels	136 (34.00%)	88 (22.00%)
Diffuse vessels	48 (12.00%)	7 (1.75%)
χ2 Value	51.6768	
*P* Value	<.0001	

### Comparison of CTA and hs-CRP in patients with DM and non-DM

Table 4 depicts the prevalence of any coronary plaque subtype according to the various hs-CRP cutoffs. Subjects with high hs-CRP levels were more likely to have any plaque (calcified plaque or mixed plaque) compared with individuals showing normal hs-CRP levels (*p*<0.01). In contrast, no significant difference was obtained for non-calcified plaque. In the study, based on the level of hs-CRP, the comparison of plaque types between DM group and non DM group showed that there was no statistical significance in non-calcified plaque with high level of hs-CRP, but the occurrence of plaque types in DM group was statistically significant compared with other hs-CRP levels in non DM group.

**Table 4:** Comparison of presence of coronary plaque between DM and non-DM patients with different hs-CRP levels

Presence of coronary plaque	DM	Non-DM	χ2 Value	*P* Value
Calcified plaque				
Low/normal hs-CRP	70 (61.4%)	94 (49.21%)	4.2668	0.0389
Intermediate hs-CRP	72 (73.47%)	50 (56.82%)	5.696	0.017
High hs-CRP	127 (67.55%)	64 (52.89%	6.7033	0.0096
Mixed plaque				
Low/normal hs-CRP	62 (54.39%)	72 (37.7%)	8.0729	0.0045
Intermediate hs-CRP	67 (68.37%)	32 (36.36%)	19.0751	< 0.0001
High hs-CRP	108 (57.45%)	52 (42.98%)	6.1749	0.013
Non-calcified plaque				
Low/normal hs-CRP	89 (78.07%)	126 (65.97%)	5.0262	0.025
Intermediate hs-CRP	84 (85.71%)	59 (67.05%)	9.0918	0.0026
High hs-CRP	144 (76.6%)	90 (74.38%)	0.1966	0.6575

### Multivariable analysis between hs-CRP and plaque characteristics in DM

Both the unadjusted and the multivariable logistic regression analyses for the presence of any coronary plaque characteristics are listed in Table 5. All DM subjects were divided into two groups according to the level of hs-CRP, and all the analyses were based on the reference category of low normal hs-CRP level group. Subjects with high hs-CRP were observed to be at increased risk for the presence of calcified plaque and severe narrowing in the unadjusted values and the adjusted model 1. No difference was observed in the risk for non-calcified plaque and mild narrowing for the unadjusted values. When examining the presence of a specific plaque characteristics with hs-CRP had no increased risk for the presence of any characteristics of plaque for the adjusted values.

**Table 5:** Multivariate logistical regression analysis of coronary plaque subtype and stenosis degree with different hs-CRP levels in DM patients

	Model	Odds ratio	Odds ratio (95% CI)
Nature of coronary plaque			
Calcified plaque	I	1.1138	0.8696–1.4266
	II	1.1347	0.8789–1.4648
Mixed plaque	I	1.0396	0.8191–1.3195
	II	0.9876	0.7709–1.2653
Non-calcified plaque	I	0.9309	0.6981–1.2413
	II	0.9311	0.6925–1.2521
Grading of stenosis			
Mild stenosis	I	0.8499	0.4691–1.5397
	II	0.8391	0.4607–1.5286
Moderate stenosis	I	1.1195	0.8779–1.4276
	II	1.0921	0.8508–1.4016
Severe stenosis	I	1.0284	0.7760–1.3619
	II	1.0112	0.7552–1.3508

Model 1: Adjusted for age and sex. Model 2: Adjusted for age, sex, smoking, low-density lipoprotein cholesterol, high-density lipoprotein cholesterol, total cholesterol, triglycerides, body mass index and DM history

## Discussion

In recent years, there has been a surge of evidence suggesting higher prevalence of cardiovascular diseases in diabetic patients. More than 75% of DM patients die from cardiovascular disease [10]. As the symptoms of coronary heart disease in DM patients are variable, this variability in symptoms often delays accurate diagnosis and subsequent treatment strategies in DM patients who are complicated with an acute coronary syndrome with poor prognosis [11]. Therefore, it is important to identify simple and efficient markers and methods to screen patient populations who have higher predilection for developing cardiovascular diseases. Coronary CTA has been a widely accepted method for non-invasive, fast, and accurate assessment of cardiovascular manifestations in DM patients.

MSCT can accurately determine the distribution, characteristics and stenosis of atherosclerosis plaques [5]. In our study, Compared with non-DM patients, the incidence of various types of plaque and lumen stenosis in DM patients was more higher than that in non-DM patients. DM patients were more likely to have more diseased vessels, especially diffuse vascular disease. Our study revealed that patients with DM had the highest rate of diffuse vascular lesions with severe stenosis and calcified plaques, which is consistent with relevant reports [12, 13]. In patients with prolonged illness, non-calcified plaques and calcified plaques were dominant, and the degree of stenosis was more severe. The extensively formed calcified plaques were likely reflective of the long-term of DM pathogenesis. Diabetic nephropathy leads to defective absorption of calcium and phosphorus, known as hyperparathyroidism, where a surge in calcium levels in the blood stream can induce excessive calcification of the arterial tube wall [14, 15]. This also suggests that progressive DM patients with coronary artery diseases are prone to acute coronary events. Coronary CTA can accurately reveal the coronary artery structures and main branches of the plaque, including the shape and density of the plaque, and the characteristics of the vascular lumen. This method is instrumentalin assessing the characteristics of the plaques and vascular lumens and help with the risk assessment of coronary heart disease.

Studies have shown that inflammatory pathways play an important role in the development of coronary atherosclerosis and thrombosis, and that hs-CRP is a reliable biomarker of inflammation [8, 16, 17]. Previous evidence also suggests that inflammation is involved in the development of DM [18, 19]. Although hs-CRP is an important cardiovascular risk factor for both healthy and coronary patients, It has been clearly demonstrated that elevated CRP levels and coronary plaque subtypes are associated with adverse cardiovascular outcomes, its clinical predictions have not yet been fully investigated [20, 21]. Understanding the relationship between CRP and vulnerable plaque and evaluating the relationship by CCTA may help to identify patients with vulnerable plaque. Studies have shown that in patients with diabetes and metabolic syndrome, hs-CRP levels are significantly increased, and the increase of hs-CRP levels is associated with insulin resistance and inflammatory responses [22, 23]. Our results compare the narrowness of vascular lumen, number of plaques and the characteristics of plaques with hs-CRP levels, Subjects with high hs-CRP levels were more likely to have any plaque compared with individuals showing normal hs-CRP levels (*p*<0.01). There was no significance statistical in non calcified plaque with high level of hs-CRP, but the occurrence of plaque types in DM group was significant statistically compared with other hs-CRP levels in non DM group. Subjects with high hs-CRP were observed to be at increased risk for the presence of calcified plaque and severe narrowing in the unadjusted values. This finding is also consistent with the relevant foreign literature reports [24, 25]. Compared with CCTA, hs-CRP is relatively safe and convenient. Although it is not possible to use it in all subjects with low risk of cardiovascular events without symptoms, the level of hs CRP in asymptomatic patients with CAD is relatively higher [26]. Therefore, people at risk for DM should pay more attention to their blood hs-CRP levels. Our findings may help understand how hs-CRP and vulnerable plaques are related, also showing that coronary CTA enables the assessment of such relationship. The combination of coronary CTA and hs-CRP might incrementally improve risk stratification in DM patients beyond conventional myocardium perfusion tests.

There are several limitations in our research. Firstly, this analysis is cross-sectional in nature, so it is impossible to make causal inference. We studied asymptomatic subjects, so our results may not apply to symptomatic individuals. Secondly, We did not include or analyze the potential effects of factors including age, gender, and smoking history on the risk of coronary heart disease in this study. Thirdly, we separated the onset of the disease into groups, but did not separate patients by the severity of their disease, or by the calcification ratings. Fourthly, is possible that our current assessment regarding the degree of stenosis is over-evaluated, which may result in no statistical significance in some analysis. We hope to further improve this aspect in future studies.

## Conclusion

Patients with DM have a higher risk of developing coronary diseases and show poor prognosis. Coronary CTA combined with hs-CRP is an effective method for detecting the cardiovascular risk factors in DM patients at early stages.

## Data Availability

The datasets generated and analysed during the current study are not publicly available due to privacy concern and patient confidentiality but are available from the corresponding author on reasonable request.

## Abbreviations

CTA: Computed tomography angiography
hs-CRP: High sensitivity C-reactive protein
DM: Diabetes mellitus
CAD: Coronary artery disease
MSCT: Multi slice computed tomography
AMI: Acute myocardial infarction
BMI: Body mass index
CPR: Curved planar reformation
RVD: Target reference vessel diameter
